# Cardiac Involvement in Eosinophilic Granulomatosis With Polyangiitis: A Retrospective Study in the Chinese Population

**DOI:** 10.3389/fmed.2020.583944

**Published:** 2020-12-10

**Authors:** Yingying Chen, Xiaoxiao Guo, Jiaxin Zhou, Jing Li, Qingjun Wu, Hongxian Yang, Shangzhu Zhang, Yunyun Fei, Wen Zhang, Yan Zhao, Fengchun Zhang, Xiaofeng Zeng

**Affiliations:** ^1^Department of Rheumatology, Peking Union Medical College Hospital, Chinese Academy of Medical Science & Peking Union Medical College, National Clinical Research Center for Dermatologic and Immunologic Diseases (NCRC-DID), Key Laboratory of Ministry of Health, Beijing, China; ^2^Department of Cardiology, Peking Union Medical College Hospital, Chinese Academy of Medical Science & Peking Union Medical College, Beijing, China; ^3^Department of Nephrology, Children's Hospital Affiliated to Capital Institute of Pediatrics, Beijing, China

**Keywords:** eosinophilic granulomatosis with polyangitis, cardiac involvement, clinical characteristics, outcome, Chinese population

## Abstract

**Introduction:** Cardiac involvement in eosinophilic granulomatosis with polyangiitis (EGPA) is associated with a poor prognosis and high mortality; however, few studies about cardiac involvement in EGPA in the Chinese population are available. We conducted this study to determine the clinical characteristics and overall outcomes of Chinese EGPA patients with cardiac involvement.

**Materials and Methods:** We retrospectively collected the clinical data of 83 patients diagnosed with EGPA and analyzed the differences between the patients with and without cardiac involvement.

**Results:** The prevalence of cardiac involvement in EGPA in this cohort was 27.7%. Compared with those without cardiac involvement, EGPA patients with cardiac involvement tended to have a younger age at onset (mean ± SD: 38.4 ± 10.5 vs. 42.1 ± 15.9 years, respectively, *p* = 0.039), higher eosinophil count (median [IQR]: 5810 [4020–11090] vs. 2880 [1530–6570] n/μL, respectively, *p* = 0.004), higher disease activity assessed using the Birmingham vasculitis activity score (BVAS) (median [IQR]: 20 [16–28] vs. 15 [12–18], respectively, *p* = 0.001), and poorer prognosis (Five Factor Score [FFS] ≥ 1: 100% vs. 38.3%, respectively, *p* = 0.001). In the cardiac involvement group, 43.5% of patients were asymptomatic, but cardiac abnormalities could be detected by cardiac examinations. With appropriate treatment, the overall outcomes of EGPA patients with cardiac involvement in our cohort were good, with only 3 (13.0%) patients dying in the acute phase and no patients dying during follow-up.

**Conclusions:** Cardiac involvement in EGPA was associated with a younger age at onset, higher eosinophil count, higher disease activity, and a poorer prognosis. Comprehensive cardiac examinations and appropriate treatment are essential to improve the prognosis of those with cardiac involvement.

## Introduction

Eosinophilic granulomatosis with polyangiitis (EGPA), formerly known as Churg-Strauss syndrome, was first described by Churg and Strauss as a rare autoimmune disease characterized by severe asthma, hypereosinophilia, and symptoms of necrotizing vasculitis in various organs (1). It is classified as antineutrophil cytoplasmic antibody (ANCA)—associated systemic vasculitis affecting small- to medium-sized vessels (2), with ANCA positivity ranging from 26.2 to 85% (3–9).

As a common target organ, cardiac involvement has been reported in 16.1–92% of patients with EGPA according to previous studies (1, 4, 9–16). The cardiac manifestations of EGPA are diverse, ranging from myocarditis, arrhythmia, valvular defects, heart failure, myocardial infarction, and pericardial effusion to cardiac tamponade (11, 13, 16–18). Cardiac involvement in EGPA strongly predicts a worse prognosis (5, 19) and higher mortality (5, 14, 20–22). To date, there have been few data available on cardiac involvement in EGPA patients in the Chinese population. We retrospectively evaluated the data of 83 Chinese patients with EGPA to analyze and describe the prevalence, clinical characteristics, prognostic factors, and outcomes of EGPA patients with cardiac involvement to increase general understanding and provide meaningful information for physicians to use during clinical practice.

## Materials and Methods

### Patients

We retrospectively collected the data of patients diagnosed with EGPA and hospitalized in Peking Union Medical College Hospital from December 2001 to June 2018 by retrieving medical records. All patients fulfilled the American College of Rheumatology criteria for the classification of EGPA (23). Briefly, patients who met four or more of the following diagnostic criteria were diagnosed with EGPA: (1) asthma, (2) eosinophilia > 10%, (3) mononeuropathy or polyneuropathy, (4) non-fixed pulmonary infiltrates, (5) paranasal sinus abnormality, and (6) extravascular eosinophils. The Birmingham vasculitis activity score (BVAS) (24) was used to assess disease activity and the revised Five Factor Score (FFS) (20) to evaluate the prognosis of all EGPA patients included in this study. According to previous studies (14, 17, 25), cardiac manifestations are attributable to EGPA when patients present with significant arrhythmia, pericardial effusion, acute heart failure, regional or global wall motion abnormalities, diastolic dysfunction, cardiomyopathy, valvular dysfunction, and myocardial enzyme abnormalities that cannot be explained by other etiologies. The study was approved by the Ethics Committee of Peking Union Medical College Hospital and was conducted in accordance with the Helsinki Declaration. Written informed consent could not be obtained due to the retrospective nature of this study.

### Clinical Data

All clinical data including age at disease onset, disease duration, initial symptoms, organ involvement, length of hospitalization, clinical manifestations, laboratory findings, imaging, treatment, and follow-up were recorded. Laboratory findings, including complete blood count, serum immunoglobulin E (IgE) levels, hypersensitive C-reactive protein (hsCRP), erythrocyte sedimentation rate (ESR), ANCA test, myocardial enzyme levels, N-terminal prohormone of brain natriuretic peptide (NT-proBNP) levels, liver function tests, and serum creatinine taken at diagnosis and after treatment were recorded retrospectively. Imaging studies included electrocardiogram (ECG), 24 hours of dynamic electrocardiogram (DCG), ultrasound, magnetic resonance imaging (MRI), computed tomography (CT), and neuroelectrophysiological examination. In addition, for patients who underwent a lung, skin, renal, or heart biopsy when indicated, the pathology results were also recorded.

### Statistical Analysis

Continuous variables are shown as the mean ± standard deviation (SD) or median (interquartile range [IQR]), while categorical variables are shown as the number (percentage). Continuous variables were analyzed using the Student's *t*-test or Mann-Whitney *U-*test, and categorical variables were analyzed using the chi-square test or Fisher's exact test, as necessary. The Type-1 error was determined to be 5%. Statistical analyses were performed using IBM SPSS statistics (Version 23.0, IBM, Armonk, NY, USA).

## Results

### Patient Data and Clinical Manifestations

Of the 83 patients with EGPA included in this study, 57 (68.7%) were men and 26 (31.3%) were women, and the mean age (± SD) was 47.3 ± 14.3 years. Twenty-three (27.7%) patients were diagnosed with cardiac involvement. The median duration before diagnosis was 36 months (IQR, 12–84 months). Sixty-four (77.1%) patients had asthma, 67 (80.7%) had paranasal sinus abnormalities, and non-fixed pulmonary infiltrates were observed in 72 (86.7%) patients. Additionally, 19 (22.9%) and 18 (21.7%) patients were diagnosed with renal and gastrointestinal involvement, respectively. Fourteen (16.9%) patients had central nervous system involvement, while 57 (68.7%) patients had manifestations of peripheral neuropathy.

For laboratory findings, all but 1 patient underwent an ANCA test. The results were positive in 26 (31.7%) patients, among which 4 (15.4%) were cANCA-positive and 22 (84.6%) were pANCA-positive. Additionally, the median absolute eosinophil count was 4160/μL (IQR, 1830–8780) for the EGPA patients in this study.

### Cardiac Involvement

**Figure 1:**
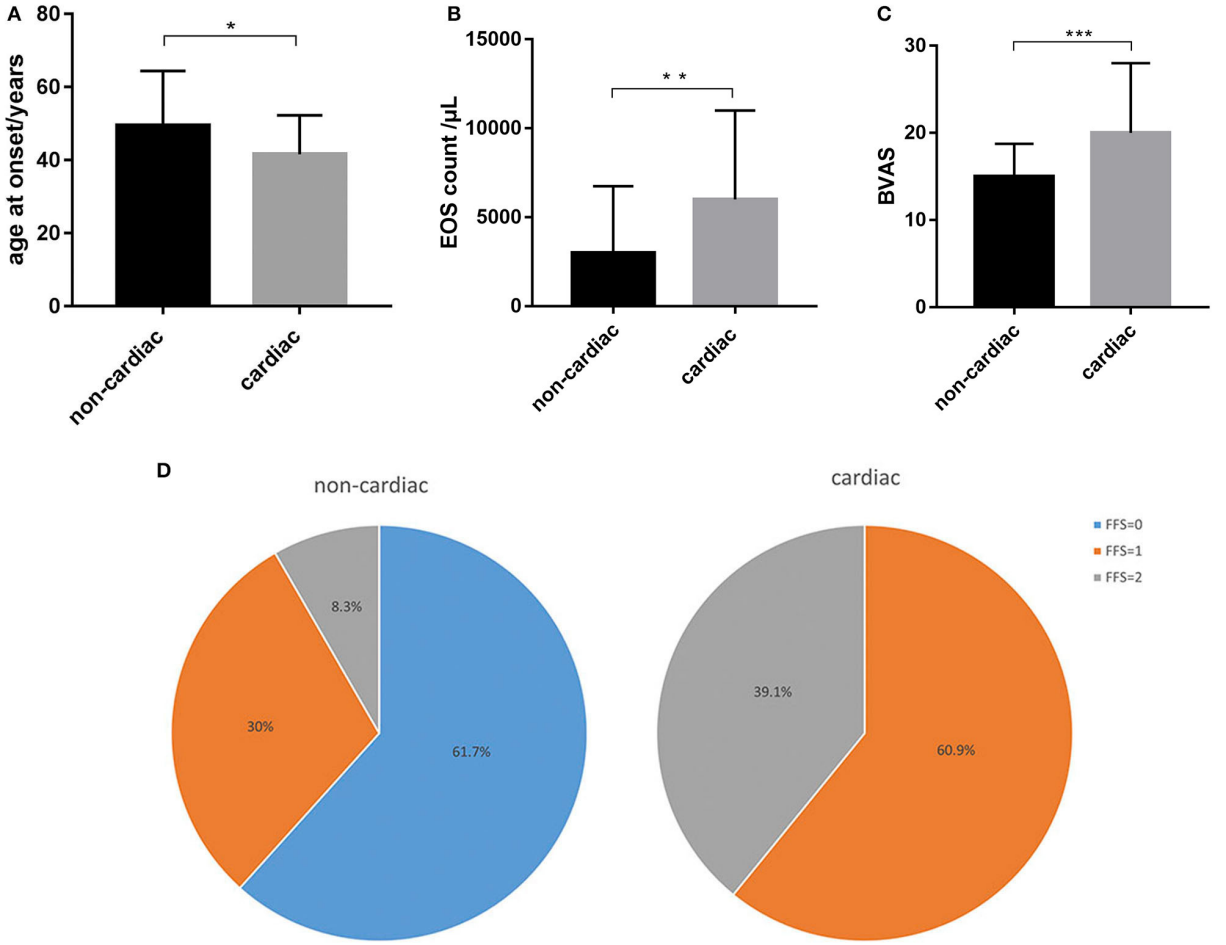
Distinct characteristics between EGPA patients with and without cardiac involvement. **(A)** EGPA patients with cardiac involvement had a younger age at onset than those without cardiac involvement. **(B)** Patients with cardiac involvement had higher eosinophil counts than those without cardiac involvement. **(C)** Patients with cardiac involvement had higher BVAS than those without cardiac involvement. **(D)** Patients with cardiac involvement had a poorer prognosis (FFS≥1) than those without. *p < 0.05, **p < 0.01, ***p < 0.001.

In this study, 23 (27.7%) patients were diagnosed with cardiac involvement, among which 13 (56.5%) presented with chest pain and dyspnea, and 10 (43.5%) were asymptomatic. A diagnosis of cardiac involvement was made using a combination of clinical symptoms, myocardial enzyme levels, ECG findings, and cardiac imaging examinations. Three (13.0%) patients were admitted to the hospital due to cardiac involvement, while the other patients were found to have cardiac involvement during hospitalization. EGPA patients with cardiac involvement had a younger age at onset than those without cardiac involvement (mean ± SD: 38.4 ± 10.5 vs. 42.1 ± 15.9 years, respectively, *p* = 0.039) (Figure 1A). Furthermore, women were more likely than men to have cardiac involvement in this cohort, since 47.8% (*n* = 11) and 25.0% (*n* = 15) in the cardiac and non-cardiac involvement group were women, respectively (*p* = 0.045). Patients with cardiac involvement tended to have higher eosinophil counts than those without cardiac involvement (median [IQR]: 5810 [4020–11090] vs. 2880 [1530–6570] /μL, respectively, *p* = 0.004) (Figure 1B). Although not significant, the percentage of ANCA positivity was lower in those with cardiac involvement than in those without (21.7 vs. 35.0%, respectively, *p* = 0.226). There were no differences between the two groups in terms of organ involvement or hsCRP, ESR, or serum IgE levels. These data are shown in detail in Table 1.

**Table 1:** Characteristics of EGPA patients with or without cardiac involvement.

	**Cardiac involvement**	**Non-cardiac**	***p*-value**
	**(*n* = 23)**	**(*n* = 60)**	
Female	11 (47.8%)	15 (25.0%)	***0.045***
Age at onset (yr)	38.4 ± 10.5	42.1 ± 15.9	***0.039***
Duration (m)	36 (24–72)	30 (12–96)	0.959
Length of hospitalization (d)	28 (20–39)	25 (20–32.8)	0.404
Follow up (m)	38 (25–69.5)	34.5 (14.3–58.5)	0.492
Clinical features			
Skin involvement	10 (43.5%)	37 (61.7%)	0.135
ENT involvement	18 (78.3%)	49 (81.7%)	0.760
Asthma	18 (78.3%)	46 (76.7%)	0.877
Lung infiltration	20 (87.0%)	52 (86.7%)	1
Renal involvement	4 (17.4%)	15 (25.0%)	0.460
GI involvement	6 (26.1%)	12 (20.0%)	0.562
Peripheral neuropathy	16 (69.6%)	41 (68.3%)	0.914
CNS manifestation	4 (17.4%)	10 (16.7%)	1
BVAS	20 (16–28)	15 (12–18)	***0.001***
FFS			
0	0 (0.0%)	37 (61.7%)	
1	14 (60.9%)	18 (30.0%)	***0.001***
2	9 (39.1%)	5 (8.3%)	
ANCApositivity	5 (21.7%)	21 (35.0%)	0.226
Eosinophil count (n/μL)	5810 (4020–11090)	2880 (1530–6570)	***0.004***
Eosinophil count (%)	42.7 (32.3–56.7)	33.3 (20.1–46.0)	***0.02***
ESR (mm/h)	30 (9–75)	40 (17.5–58.5)	0.962
hsCRP (mg/L)	25.2 (7.1–81.3)	29.3 (9.2–98.5)	0.755
Serum IgE (kU/L)	469 (212.5–1635)	413.5 (162.5–1524.3)	0.681

*ANCA, antineutrophil cytoplasmic antibody; BVAS, Birmingham vasculitis activity score; CNS, central nervous system; ENT, ears, nose, and throat; ESR, erythrocyte sedimentation rate; FFS, Five Factor Score; GI, gastrointestinal; hsCRP, hypersensitive C-reactive protein; IgE, immunoglobulin E. Bold and italic form to highlight the p values that are significant*.

All patients with cardiac involvement underwent an ECG test. Four (17.4%) were found to have sinus tachycardia and 5 (21.7%) had ST-segment or T wave abnormalities. One (4.3%) patient with ST-segment elevation progressed to cardiac arrest but survived due to timely cardio-pulmonary resuscitation. One (4.3%) patient was found to have a third-degree atrioventricular block requiring support from a temporary pacemaker. Ten (43.5%) patients did not present with ECG abnormalities but had elevated myocardial enzymes or echocardiographic changes. Thirteen patients (56.5%) had elevated cardiac Troponin I (cTn I) levels, and elevated NT-proBNP levels were found in 12 patients (52.2%).

An echocardiography was performed on all patients with cardiac involvement, and 21 (95.5%) patients were found to have cardiac disorders. Six (26.1%) patients had decreased left ventricular ejection fraction (LVEF; median [IQR]: 34.1% [31%−46%]), among which one patient had extremely low LVEF (11%), leading to cardiogenic shock and the need for extracorporeal membrane oxygenation support. Eleven (47.8%) patients had diastolic function abnormalities or segmental dyskinesia, and 6 (26.1%) patients had mitral or tricuspid insufficiency. Mild to moderate pericardial effusion was observed in 7 (30.4%) patients. Only 2 (8.7%) patients underwent a cardiac MRI. Both of these patients were found to have late gadolinium enhancement of the myocardium and one had decreased LVEF (43.8%) which was not identified on echocardiography. None of the 6 patients who underwent a coronary artery CT angiography (CTA) had major abnormalities that could be characterized as cardiac involvement. These findings are shown in Table 2.

**Table 2:** Cardiac findings, treatment, outcome and follow-up of 23 patients with EGPA.

**Case**	**Age/sex**	**cTn I**	**NT-proBNP**	**ECG**	**CTA**	**Echocardiography**	**Cardiac**	**BVAS**	**FFS**	**Induction**	**Maintenance**	**Outcome**	**Follow-up**
							**MRI**			**therapy**	**therapy**		
1	40/F	NA	NA	Sinus tachycardia	NA	LVEF 37.2%	NA	34	2	MP pulse/CTX	-	Death	-
2	34/M	↑	↑	Left anterior hemiblock	Normal	LVEF46%, diastolic function abnormality	NA	15	1	MP pulse/CTX	MP	Remission	Remission
3	39/F	↑	↑	Sinus tachycardia	Normal	Normal	NA	16	1	MP pulse/CTX	pred/CTX	Remission	Relapse
4	37/M	Normal	Normal	ST-segment elevation	25% stenosis of LAD	LVEF 46%, segmental dyskinesia	NA	13	2	MP/CTX	pred/CTX	Remission	Relapse
5	49/M	Normal	NA	Normal	NA	Echo-enhancement of myocardium, hypokinesia	NA	29	2	MP pulse/CTX	MP/CTX	Remission	Remission
6	25/M	NA	NA	Normal	NA	Pericardial effusion	NA	16	1	pred/CTX	pred	Remission	Lost
7	49/F	↑	↑	AVB grade III	NA	LVEF 31%, diastolic function abnormality	NA	24	1	MP/CTX	MP/CTX	Remission	Remission
8	36/F	↑	NA	PVC	NA	LVEF 31% → 11%, mitral insufficiency	NA	18	2	MP pulse/CTX	pred/CTX	Remission	Remission
9	49/F	↑	↑	Normal	Normal	Pericardial effusion, mitral and tricuspid insufficiency	NA	20	1	MP/CTX	MP/CTX	Remission	Remission
10	34/F	↑	NA	Normal	NA	Normal	NA	19	2	MP/CTX	pred/CTX	Remission	Remission
11	30/F	↑	↑	Normal	NA	Mild mitral insufficiency	NA	14	1	MP pulse/CTX	pred/CTX	Remission	Remission
12	42/F	↑	↑	Normal	Normal	Myocardiopathy, hypokinesia	NA	21	1	MP pulse/CTX	pred/CTX	Remission	CHF
13	48/M	Normal	Normal	Normal	NA	Echo-enhancement of left ventricular apex, hypokinesia	NA	24	1	MP/CTX	MP/CTX	Remission	Remission
14	26/M	↑	↑	V1-6 ST-segment depression	NA	LVEF 31%, mitral insufficiency, hypokinesia, mild PAH	NA	38	2	MP pulse/CTX	-	Death	-
15	57/M	Normal	↑	PVC, SVT	NA	LVEF 68%, hypokinesia, mild pericardial effusion	NA	19	1	MP pulse/CTX	pred/CTX	Remission	Remission
16	54/F	↑	↑	Normal	NA	Pericardial effusion	NA	33	2	MP pulse/CTX	-	Death	-
17	28/M	Normal	Normal	Normal	NA	LVEF 63%, segmental dyskinesia	LGE of interventricular septum	20	1	pred/CTX	pred/CTX	Remission	Remission
18	34/M	Normal	↑	Sinus tachycardia	NA	LVEF 50%, hypokinesia, mild pericardial effusion	NA	42	2	MP/CTX	pred/CTX	Remission	Remission
19	31/F	Normal	Normal	sinus tachycardia, I, II, III, aVF, V4-6 ST-segment depression	NA	LVEF 73%,diffuse thickening of pericardium, mild pericardial effusion	NA	12	1	MP/CTX	pred/CTX	Remission	Relapse
20	55/F	↑	↑	V1-5 T wave inversion	NA	LVEF 62%, mild pericardial effusion	NA	18	1	MP/CTX	pred/CTX	Remission	Remission
21	57/M	Normal	Normal	P mitrale	NA	Mitral insufficiency, mild PAH	NA	16	1	MP pulse/CTX	MP/CTX	Remission	Remission
22	53/M	↑	↑	Normal	Normal	LVEF 62%, segmental dyskinesia	LGE of left and right ventricular wall, LVEF=43.8 %, mild pericardial effusion	23	1	MP pulse/CTX	MP/CTX	Remission	Remission
23	53/M	↑	NA	V4-6 ST-segment depression	NA	Left ventricular hypertrophy, mitral insufficiency	NA	28	2	MP pulse/CTX	MP/CTX	Remission	Remission

*AVB, atrioventricular block; CHF, chronic heart failure; CTA, computed tomography angiography; cTn I, cardiac Troponin I; CTX, cyclophosphamide; ECG, electrocardiogram; LAD, left anterior descending; LGE, late gadolinium enhancement; LVEF, left ventricular ejection fraction; MP, methylprednisolone; MRI, magnetic resonance imaging; NA, not available; NT-proBNP, N-terminal prohormone of brain natriuretic peptide; PAH, pulmonary artery hypertension; pred, prednisone; PVC, premature ventricular contraction; SVT, supraventricular tachyca*.

### Disease Activity, Treatment, Outcomes, and Follow-Up

The patients with cardiac involvement had higher BVASs than those without cardiac involvement (median [IQR]: 20 [16–28] vs. 15 [12–18], respectively, *p* = 0.001), which indicated greater disease activity in the cardiac involvement group (Figure 1C). Moreover, patients with cardiac involvement tended to have a poorer prognosis than those without (FFS ≥1: 100 vs. 38.3%, respectively, *p* = 0.001) (Figure 1D).

All patients with cardiac involvement received a combination of glucocorticoids and cyclophosphamide (CTX) as induction treatment. The methylprednisolone pulse therapy involved intravenous methylprednisolone 1g per day for 3 days followed by high dose glucocorticoids. High-dose glucocorticoid treatment was defined as oral or intravenous glucocorticoids equivalent to prednisone 1–2 mg/(kg·d). Fifteen (65.2%) patients received methylprednisolone pulse in addition to CTX therapy, and 8 (34.8%) received high-dose glucocorticoids in addition to CTX. Maintenance treatment included glucocorticoids and CTX (*n* = 18) or glucocorticoids alone (*n* = 2). In terms of the outcomes for the EGPA patients with cardiac involvement, one patient died of infectious shock due to gut perforation, one patient died of multi-organ failure, and one died from a cerebral hernia. Twenty (87.0%) patients achieved remission after treatment, which included an improvement in LVEF, a decrease in myocardial enzymes, and/or a normal ECG.

For the patients with cardiac involvement, the mean follow-up period was 38 months (IQR, 25–69.5), and one patient was lost to follow-up. Three (15.8%) patients relapsed and were readmitted to the hospital during the follow-up period. The relapsed organs for these patients were the heart, ear, and lung, and all patients achieved remission after treatment. No relationship was observed between relapsing and disease activity, which was measured using the BVAS or FFS at baseline. One patient (5.3%) progressed to heart failure, 15 (78.9%) patients achieved long-term remission, and no patients died during follow-up. These findings are shown in Table 2.

## Discussion

EGPA is believed to have a better prognosis than other types of ANCA-associated vasculitis (5, 26), though cardiac involvement is an independent risk factor of mortality (5, 20). Previous studies have demonstrated a prevalence of cardiac involvement in EGPA ranging from 16.1 to 92%, while the prevalence in our cohort was 27.7%, which was relatively low. One reason may be that only two patients underwent a cardiac MRI, which is more sensitive than other imaging studies and can identify cardiac abnormalities in EGPA patients even after a normal ECG and echocardiography as previous studies have suggested (9, 14, 27–29). Patients with cardiac involvement had characteristics that were distinct from those without cardiac involvement in this cohort. According to previous studies (11, 12, 17, 30), EGPA is more common in males than in females, which was also observed in our cohort (68.7 vs. 31.3%, respectively). However, there were more female patients (47.8%) in the cardiac involvement group than in the non-cardiac involvement group (25.0%), indicating that female patients had a higher incidence of cardiac involvement in this population. Furthermore, patients with cardiac involvement in our study tended to have a younger age at onset compared with those without cardiac involvement. This finding was not identified in other studies. Many studies have demonstrated that ANCA negativity is correlated with heart and lung involvement (4, 6, 19, 25), while ANCA positivity is correlated with renal involvement and pulmonary hemorrhage (4, 6, 31). Thus, it has been suggested that cardiac involvement in EGPA is the result of eosinophilic infiltration in the tissue (1, 32), and that eosinophilia plays a more significant role than ANCA in EGPA with cardiac involvement (15). In our study, we also noted a higher eosinophil count in the group with cardiac involvement, which was consistent with the findings of previous studies (15, 25). The percentage of ANCA positivity was lower in the group with cardiac involvement in this study, although this finding was not statistically significant. Many of the patients had taken glucocorticoids before diagnosis, which may explain this phenomenon.

A diagnosis of cardiac involvement was made using a combination of clinical manifestations; myocardial enzyme levels; and ECG, coronary artery CTA, echocardiography, and cardiac MRI findings. Chest pain was a common symptom in patients with cardiac involvement, which is in accordance with some previous studies (15, 33). However, almost half of the patients were asymptomatic or had normal ECGs, and cardiac abnormalities were only detected by echocardiography or cardiac MRI. Therefore, to exclude cardiac involvement even after a normal ECG and the absence of cardiac manifestations, an overall evaluation of the patient's heart condition should be performed, as recommended by previous studies (14, 17), especially in patients with an early age of onset and a high eosinophil count according to the results of our study. EGPA patients with cardiac involvement have been reported to have higher FFSs (9, 25), which was also noted in our cohort, suggesting that patients with cardiac involvement have a poorer prognosis. Additionally in our study, patients with cardiac involvement had higher BVASs, which predicts greater disease activity and severe organ damage. However, the application of glucocorticoids and the combination of immunosuppressive agents such as CTX in severe cases significantly improved the outcomes for EGPA (5, 19). All patients with cardiac involvement in our study received the induction treatment, which included methylprednisolone pulse therapy or high-dose glucocorticoids combined with CTX, and most patients achieved remission during the acute phase. Due to this intensive therapy, the overall outcomes of patients with cardiac involvement in our cohort were good, similar to the results of some previous studies (25, 34). Therefore, early diagnosis and appropriate treatment are essential to prevent the acceleration of cardiac involvement in patients with EGPA (17, 34, 35). Recently, mepolizumab, a fully humanized monoclonal antibody that blocks IL-5 (36), was approved by the US Food and Drug Administration for use in EGPA. Mepolizumab, which could lead to a significant decrease in peripheral absolute eosinophil counts, has been effective in EGPA treatment (37–40), leading to longer periods of remission and reduced doses of glucocorticoids. Since cardiac involvement in EGPA has been correlated with a high peripheral eosinophil count, mepolizumab could be an effective treatment for EGPA with cardiac involvement.

This is the first study to our knowledge with a relatively large sample size addressing EGPA patients in China with cardiac involvement. We identified that the clinical characteristics of patients with cardiac involvement are distinct from those of patients without cardiac involvement in this cohort, which may provide meaningful information for physicians to use during clinical practice. Moreover, our study suggests that cardiac involvement predicts a poorer prognosis and higher disease activity in EGPA patients. Comprehensive cardiac examinations and appropriate treatments are essential to diagnose cardiac involvement early, thus improving outcomes for this patient population.

However, our study has several limitations. First, this is a cross-sectional and retrospective study, so biases may have influenced the results. Additionally, the fact that endomyocardial biopsies were not performed to confirm the diagnosis of cardiac involvement is another limitation of our study.

In conclusion, EGPA patients with cardiac involvement tended to have a younger age at onset, higher eosinophil count, higher disease activity, and a poorer prognosis than those without cardiac involvement. Comprehensive cardiac examinations are therefore necessary to diagnose cardiac involvement early. High-dose glucocorticoids combined with CTX was an effective treatment used to prevent the acceleration of cardiac involvement.

## Data Availability Statement

The raw data supporting the conclusions of this article will be made available by the authors, without undue reservation.

## Ethics Statement

The studies involving human participants were reviewed and approved by Ethics Committee of Peking Union Medical College Hospital. Written informed consent for participation was not required for this study in accordance with the national legislation and the institutional requirements.

## Author Contributions

YC collected and analyzed the data and wrote the manuscript. JZ, JL, QW, and HY collected and analyzed the data and revised the manuscript. XG, WZ, YZ, FZ, and XZ designed the study, re-confirmed the diagnoses of all the patients, and revised the manuscript. SZ and YF designed the study, analyzed the data, and wrote the manuscript. All authors have read and approved the final manuscript.

## Conflict of Interest

The authors declare that the research was conducted in the absence of any commercial or financial relationships that could be construed as a potential conflict of interest.
